# Aerobic Exercise for a Duration of 90 min or Longer Per Week may Reduce the Atherogenic Index of Plasma

**DOI:** 10.1038/s41598-018-20201-x

**Published:** 2018-01-29

**Authors:** Shiwei Shen, Huajin Qi, Xingxian He, Yun Lu, Chengjian Yang, Feng Li, Ling Wang, Dongchang Qiang, Kedong Shui, Lin Zhou, Xiaofeng Weng, Zhenhai Shen, Liuxin Wu

**Affiliations:** 1 Wuxi No.2 People’s Hospital Affiliated to Nanjing Medical University, Wuxi, Jiangsu 214002 China; 2 The Taihu Rehabilitation Hospital of Jiangsu Province (Jiangsu Provincial Research Center for Health Assessment and Intervention), Wuxi, Jiangsu 214086 China; 3 The Nanchansijiedao Community Health Service Center, Wuxi, Jiangsu 214007 China; 4 Zhongguancun Xinzhiyuan Health Management Institute, Beijing, 100142 China

## Abstract

The correlation between the weekly duration of aerobic exercise and atherogenic index of plasma (AIP) is still unknown. A cross-sectional study was conducted involving 27,827 middle-aged Chinese men who had health examinations in our hospital. The correlation between the duration of moderate-intensity aerobic exercise and AIP was determined. The mean AIP levels were 0.1166 ± 0.34475, 0.1167 ± 0.32637, and 0.0765 ± 0.32872 in the non-exercise (PA1), occasional exercise (PA2), and frequent exercise groups (PA3), respectively. Significantly higher AIP levels were observed in the PA1 and PA2 groups than the PA3 group, while no significant difference existed between the PA1 and PA2 groups. Physical activity significantly reduced the AIP after adjustment for age, body mass index, diastolic blood pressure, and fasting blood glucose and uric acid levels. In addition, the percentage of the population at high risk for atherosclerosis (AIP ≥ 0.21) was significantly lower in the PA3 group than the PA1 and PA2 groups. Moderate-intensity aerobic exercise at a weekly duration of 90 min or longer is associated with the reduction of AIP among middle-aged men in southeastern China.

## Introduction

Cardiovascular disease is a global public health concern^1,2^. Greater than 290 million people are estimated to have cardiovascular diseases in China^3^. Chinese people have a lower rate of physical exercise, and the amount of physical exercise is trending down^4^. It has been established that regular physical activity reduces the risk of developing cardiovascular diseases^5–10^. Therefore, the American Heart Association (AHA) included physical activity as one of the seven indicators for ideal cardiovascular health^11^. As an indirect parameter indicative of the size of low-density lipoprotein (LDL) particles, the atherogenic index of plasma (AIP) is an indicator that measures the degree of atherosclerosis, which is of value in the evaluation of risk for human arteriosclerotic cardiovascular disease^12^. Recently Edwards MK *et al*.^13^ used accelerometry data from NHANES and showed that higher physical activity is associated with lower AIP. Our previous studies^14^ have found the ideal cardiovascular health score correlated significantly with AIP, and a 1-point increase in the cardiovascular health score led to a 0.046 reduction in AIP. We also found^15^ weight control combined with increased aerobic exercise time may cause a synergistic effect on the reduction of AIP. The major purpose of the present study was to determine the correlation between the weekly duration of physical activity and AIP among middle-aged men in southeastern China to provide evidence for the development of preventive and control strategies for cardiovascular diseases.

## Methods

### Subjects

A cross-sectional study was conducted. Detailed methods can be seen in ref.^15^, in which 1126 cases without waist circumference data were excluded, while in this study, as waist circumference is not required, these 1126 cases were included. Therefore a total of 27,827 Chinese men between 40 and 64 years of age were enrolled in this study.

The study protocol was approved by the Ethics Review Committee of the Taihu Rehabilitation Hospital of Jiangsu Province, and the study was performed in accordance with the principles of the Declaration of Helsinki. Written informed consent was obtained from all participants following a detailed description of the purpose of this study.

Questionnaire survey and Measurements of cardiovascular risk factors were obtained according to the method in ref.^15^.

The atherogenic index of plasma (AIP) is defined as AIP = log_10_ (TG/HDL-C)^16^.

Physical activity was defined as moderate-intensity aerobic exercise, and included fast walking, running, bicycle riding, rope skipping, and swimming. All subjects were assigned to three groups (1) non-exercise group (PA1 group), no extra physical activity except daily life and work activities; (2) occasional exercise group (PA2 group), physical activity <three times a week and <30 min each session or <90 min of physical activity a week; and (3) frequent exercise group (PA3 group), physical activity >three a week and >30 min each session or >90 min a week.

AIP was also assigned to four groups according to quartile (Q1, Q2, Q3, and Q4) in each PA groups, respectively. And difference in the distribution of AIP quartiles among the each groups were compared.

### Risk grouping of atherosclerosis

All participants were assigned to one of three groups according to the AIP level. Subjects with an AIP < 0.11, an AIP between 0.11 and 0.21, and an AIP > 0.21 were assigned to the low-, intermediate, and high-risk atherosclerosis groups, respectively^9,17^.

### Statistical analysis

All measurement data are presented as the mean ± standard deviation (SD), while count data are expressed as the number (proportion). Differences in proportions were tested for statistical significance using a chi-square test, and the group comparisons were analyzed by the method of Least Significant Difference (LSD) in ANOVA. The factors affecting AIP were identified using linear regression analysis. All statistical analyses were performed using SPSS (version 16.0; SPSS, Inc., Chicago, IL, USA), with a *P* value <0.05 considered statistically significant.

## Results

### Comparison of baseline cardiovascular risk factors among groups

A total of 27,827 Chinese middle-aged men were enrolled in this study, with a mean age of 50.78 ± 6.758 years. Significant differences existed in SBP, DBP, FBP, TC, TG, HDL-C, LDL-C, UA, and AIP among the PA1, PA2, and PA3 groups; however, no significant difference was detected in the BMI among the three groups (Table 1).

**Table 1:** Comparison of baseline cardiovascular risk factors among groups.

Group	n	BMI (kg/m^2^)	SBP (mmHg)	DBP (mmHg)	FPG (mmol/L)	TC (mmol/L)	TG (mmol/L)	HDL-C (mmol/L)	LDL-C (mmol/L)	UA (μmol/L)	AIP
PA1	2657	25.2 ± 3.15	132 ± 17.5	82 ± 11.3	5.63 ± 1.243	4.92 ± 0.894	2.05 ± 1.902	1.28 ± 0.307	2.91 ± 0.747	366 ± 80.5	0.1166 ± 0.34475
PA2	12471	25.1 ± 2.92	131 ± 16.6	81 ± 10.8	5.52 ± 1.083	4.86 ± 0.862	1.99 ± 1.719	1.27 ± 0.301	2.85 ± 0.720	372 ± 76.2	0.1167 ± 0.32637
PA3	12699	25.0 ± 2.93	132 ± 16.6	82 ± 10.7	5.56 ± 1.052	4.83 ± 0.820	1.84 ± 1.595	1.29 ± 0.302	2.84 ± 0.695	367 ± 75.9	0.0765 ± 0.32872
F		1.753	19.445	6.629	11.925	16.968	31.027	14.419	11.136	12.583	51.286
p		0.173	<0.001	0.001	<0.001	<0.001	<0.001	<0.001	<0.001	<0.001	<0.001

BMI, indicates body mass index; SBP, systolic blood pressure; DBP, diastolic blood pressure; FPG, fasting plasma glucose; TC, total cholesterol; TG, triglycerides; HDL-C, high-density lipoprotein cholesterol; LDL-C, low-density lipoprotein cholesterol; UA, uric acid; AIP, atherogenic index of plasma;

PA1 group, non-exercise group, no extra physical activity except daily life and work activities

PA2 group, occasional exercise group, physical activity <three times a week and <30 min each session or <90 min of physical activity a week; PA3 group, frequent exercise group, physical activity >three a week and >30 min each session or >90 min a week.

### Comparison of AIP among groups

The mean AIP of all subjects was 0.0984 ± 0.32983. Significantly higher mean AIP levels existed in the PA1 (0.1166 ± 0.34475) and PA2 groups (0.1167 ± 0.32637) compared to the PA3 group (0.0765 ± 0.32872), while no significant difference was noted between the PA1 and PA2 groups (Table 2).

**Table 2:** Comparison of AIP among groups.

	AIP	Group Comparison	Mean difference	S.E.	P	95% CI
PA1	0.1166 ± 0.34475	PA1 & PA2	−0.00010	0.00703	0.989	−0.0139~0.0137
PA2	0.1167 ± 0.32637	PA1 & PA3	0.04005	0.00702	< 0.001	0.0263~0.0538
PA3	0.0765 ± 0.32872	PA2 & PA3	0.04015	0.00415	< 0.001	0.0320~0.0483

PA1 group, non-exercise group, no extra physical activity except daily life and work activities

PA2 group, occasional exercise group, physical activity <three times a week and <30 min each session or <90 min of physical activity a week

PA3 group, frequent exercise group, physical activity >three a week and >30 min each session or >90 min a week.

### Multivariate linear regression analysis of factors affecting AIP

Age, BMI, SBP, DBP, FPG, UA, and physical activity were included in the linear regression model (backward method), while AIP served as a dependent variable. Multivariate linear regression analysis revealed that age(B = −0.003), BMI(B = 0.019), DBP(B = 0.002), FPG(B = 0.046), UA(B = 0.001), and physical activity(−0.022) affected AIP (all P < 0.001).

### Distribution of AIP quartiles among the groups

**Figure 1:**
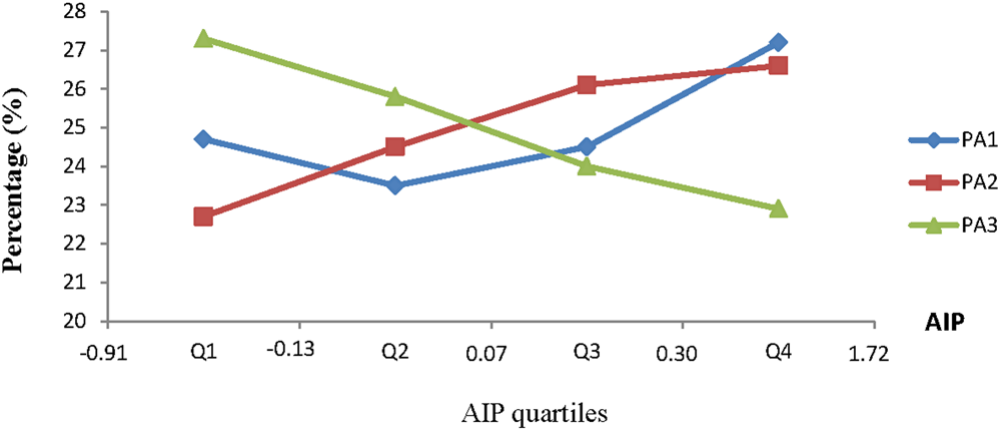
Distribution of AIP quartiles among the groups. PA, Physical Activity. PA1 group, non-exercise group, no extra physical activity except daily life and work activities; PA2 group, occasional exercise group, physical activity <three times a week and <30 min each session or <90 min of physical activity a week; PA3 group, frequent exercise group, physical activity >three a week and >30 min each session or >90 min a week. AIP, Atherogenic Index of Plasma Q1, 1st quartile, n = 6959, AIP(−0.19~−0.13); Q2, 2^nd^ quartile, n = 6956, AIP(−0.13~0.07); Q3, 3^rd^ quartile, n = 6955, AIP(0.07~0.30); Q4, 4^th^ quartile, n = 6957, AIP(0.30~1.72).

AIP was assigned to four groups according to quartile (Q1, Q2, Q3, and Q4), as follows: 24.7%, 23.5%, 24.5%, and 27.2% in the PA1 group; 22.7%, 24.5%, 26.1%, and 26.6% in the PA2 group; and 27.3%, 25.8%, 24.0%, and 22.9% in the PA3 group, respectively. There was a significant difference in the distribution of AIP quartiles among the PA1, PA2, and PA3 groups (Fig. 1).

### Correlation between the amount of aerobic exercise and atherosclerosis risk

The proportions of subjects at low-, intermediate-, and high-risk for atherosclerosis were 53.5%, 11.5%, and 35.0% in the PA1 group, 52.5%, 12.5%, and 34.9% in the PA2 group, and 58.0%, 11.6%, and 30.4% in the PA3 group, respectively, and there was a significant difference among the three groups (Table 3).

**Table 3:** Relationship between aerobic exercise and atherosclerosis risk (%).

Group	low-risk	intermediate-risk	high-risk	χ^2^	P
PA1	53.5	11.5	35.0	84.529	< 0.001
PA2	52.5	12.5	34.9		
PA3	58.0	11.6	30.4		

## Discussion

There is an increase in the number of sedentary populations worldwide, which contributes to general health conditions and the prevalence of non-communicable chronic diseases. Lack of physical activity has been identified as the fourth leading cause of death worldwide^18^. Data from the China Health and Nutrition Survey (CHNS) showed a remarkable downward trend in the amount of physical activity among Chinese people between 18 and 49 years of age, and the total amount of physical activity decreased 29% and 38% in men and women in 2009, respectively, relative to 1997^19^. Considering the importance of physical activity in public health, the World Health Organization developed the “Global Recommendations on Physical Activity for Health” in 2010, with the core aim of achieving primary prevention of non-communicable chronic diseases through promotion of physical activity^18^. In 2011, the Chinese Center for Disease Control and Prevention proposed the “Guidelines on Physical Activity for Chinese Adults,” with aims to facilitate the implementation of physical activity and improve national physical quality and health level^20^, thereby resulting in the prevention and control of chronic diseases.

Aerobic exercise is a type of physical activity involving large muscles for a long duration, and is widely preferred by middle-aged people. Aerobic exercise is reported to decrease fasting or postprandial TG and increase HDL-C by improving the concentration and activity of lipoprotein lipase (LPL) in skeletal muscles and accelerating lipid transfer, decomposition, and excretion^21^. Our findings showed a significantly lower TG level in the PA3 group than the PA1 and PA2 groups, and the HDL-C concentration was significantly greater in the PA3 group relative to the PA1 and PA2 groups. It has been demonstrated that 8–14 weeks of aerobic exercise leads to a decline in fasting TG level by 4–37%, and an increase in HDL-C concentration by 4–18%^22^. A weekly energy consumption ≥ 1000 kcal has been shown to decrease the TG level in men^23^, and a weekly energy consumption ≥900 kcal or >120 min of exercise a week has been reported to significantly increase HDL-C concentration, which is not affected by exercise intensity^24^. Our findings showed a significantly lower mean AIP in frequent exercisers (PA3 group) than non-exercisers (PA1 group) and occasional exercisers (PA2 group), and there was no significant difference in the mean AIP between the PA1 and PA2 groups. In addition, physical activity was shown to reduce the AIP after the adjustment of age, BMI, DBP, and FPG and UA levels.

AIP has been shown to be negatively correlated with the diameter of LDL-C particles, and has been recommended as an indirect parameter to measure the size of LDL-C particles^8^. An increase in AIP indicates a decrease in the diameter of LDL-C particles, and an increase in the proportion of small dense LDL (sdLDL)^6^. sdLDL, a subtype of LDL components that may cause atherosclerosis, has been identified as a major risk factor for coronary heart diseases and was recommended for detection by the National Cholesterol Education Program in 2002^25^. The currently available methods for detection of sdLDL have limitations, which have limited the clinical application. Therefore, AIP has superiority in the assessment of the risk for human atherosclerosis. The results of the current study showed a decreasing trend of the constituent ratio of Q1, Q2, Q3, and Q4 in the PA3 group and an increasing trend in the constituent ratio of Q1, Q2, Q3, and Q4 in the PA1 and PA2 groups; no significant difference was shown in the distribution of AIP quartiles between the PA1 and PA2 groups. In addition, the proportion of subjects at high risk for atherosclerosis was significantly lower in the PA3 group than the PA1 and PA2 groups, and the proportion of subjects at low risk for atherosclerosis was significantly higher in the PA3 group than the PA1 and PA2 groups, while no significant difference was observed between the PA1 and PA2 groups. The results demonstrated that the AIP is comparable in subjects with <90 min of physical activity weekly and non-exercisers, and a significantly higher AIP was shown in subjects with >90 min of physical activity weekly and ≥90 min of physical activity weekly. The excess post-exercise oxygen consumption of aerobic exercise may be sustained for ≥48 h^23,26,27^. In addition, the American College of Sports Medicine recommends at least 3–5 exercise sessions per week, and the health benefit from 1–2 exercise sessions per week was less than 3–5 exercise sessions per week; however, comparable health benefits existed between subjects engaged in exercise daily and subjects exercising five times per week. The study subjects enrolled in this study were middle-aged Chinese men whose living conditions, dietary habits and constitution differ from Western populations. Our findings indicate that moderate-intensity aerobic exercise ≥ three times per week and an accumulated duration ≥90 min a week may achieve substantial benefits with respect to cardiovascular health.

### Limitations

The amount of physical activity was calculated using the duration of activity, which was not transformed to energy consumption. In addition, the study subjects recruited in this study were middle-aged Chinese men. Therefore, region-, race-, gender-, and age-specific variations should be taken into account if the conclusions drawn from this study are used.

## Conclusions

Physical activity has multiple health benefits, including weight loss, lowering of blood pressure, and improvement in blood lipid and glucose levels. The AHA highlights the significance of physical activity in terms of cardiovascular health. Our findings showed that weekly moderate-intensity aerobic exercise ≥90 min significantly might decrease the AIP and other cardiovascular risk factors in middle-aged men. Given that a lack of physical activity is a major risk factor for cardiovascular diseases in China, physical activity-based health promotion may be a low-cost, highly-effective strategy for the promotion, prevention, and control of cardiovascular health.
